# Supplementary figures and images for: Intratumoral Microbiota Correlates with AP-2 Expression: A Pan-Cancer Map with Cohort-Specific Prognostic and Molecular Footprints
Source: Int J Mol Sci. 2025 Nov 29;26(23):11587. doi: 10.3390/ijms262311587 (PMC12692072; doi:10.3390/ijms262311587)

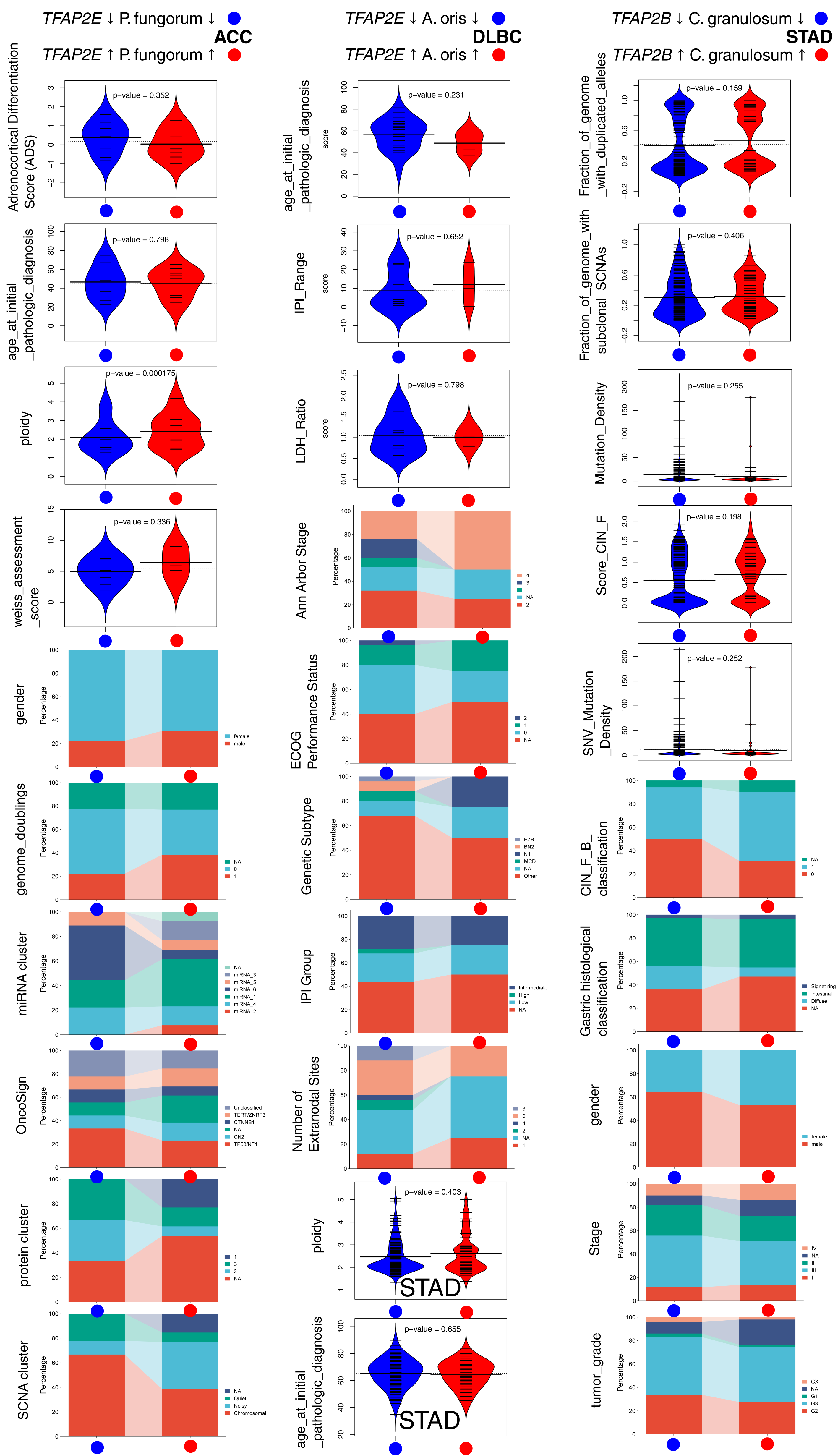

Supplement: Supplementary file 1 [file ijms-26-11587-s001.zip › Figure S1.tif]

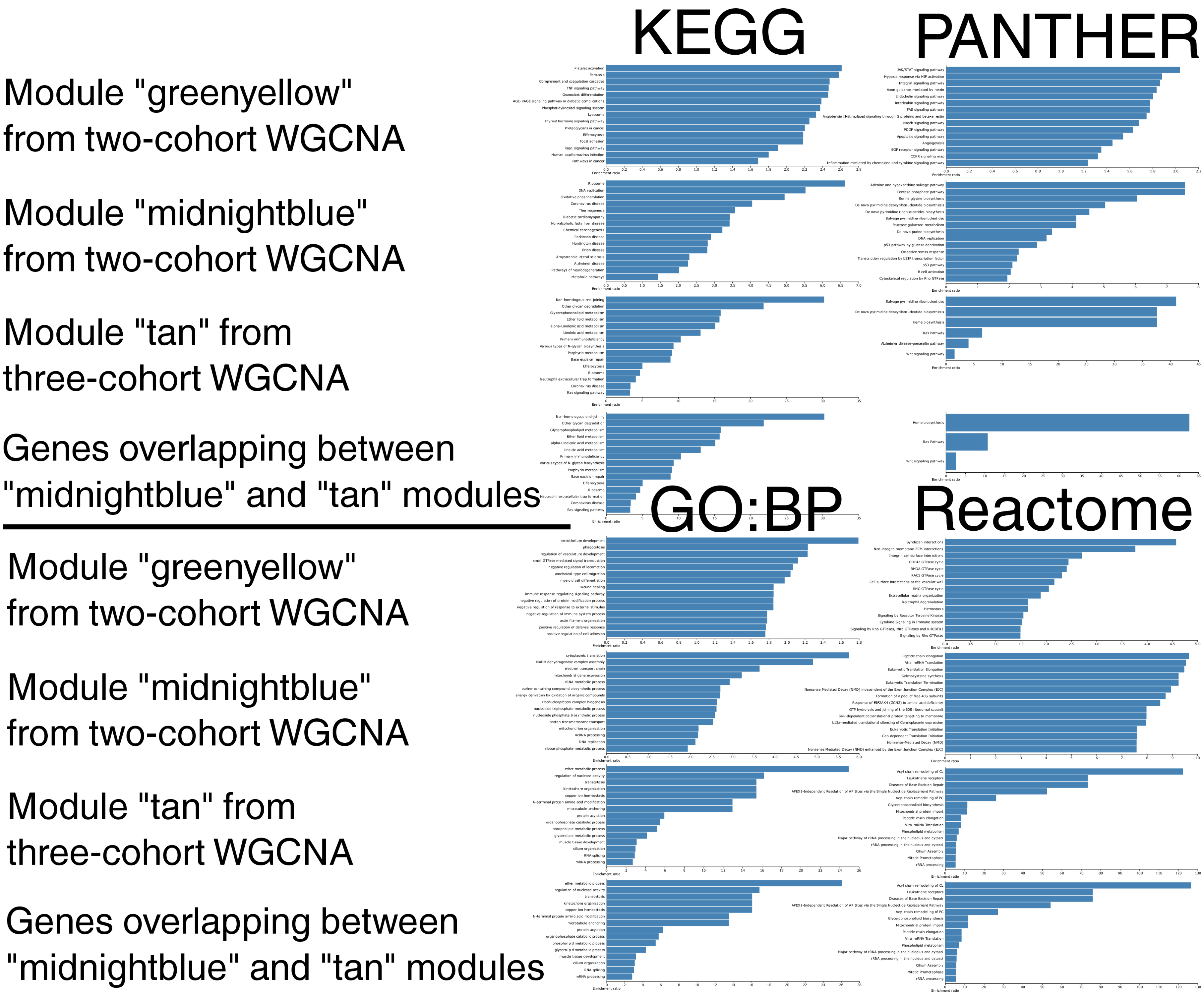

Supplement: Supplementary file 1 [file ijms-26-11587-s001.zip › Figure S2.tif]
